# Smooth muscle relaxant activity of *Crocus sativus* (saffron) and its constituents: possible mechanisms

**Published:** 2018

**Authors:** Mansoureh Gorginzadeh, Mansoureh Vahdat

**Affiliations:** *Endometriosis Research Center, Iran University of Medical Sciences, Tehran, Iran*

**Keywords:** Saffron, Crocus sativus, Muscle fibers, Uterus, Cervical tissue

**Dear editor**

We praise the article by Mokhtari-Zaer et al. (2015) ▶ published in the Avicenna Journal of Phytomedicine on the effects of saffron on smooth muscle activity (Mokhtari-Zaer et al., 2015 ▶). It was a well-designed and interesting study on the therapeutic properties of *Crocus sativus* which is a precious plant cultivated in our country. We do believe that along with all other medicinal effects, saffron or its active constituents, primarily crocin and crocetin, could also have some clinical implications in the gynecology field. Historically and particularly in traditional Persian medicine, saffron has been regarded as an abortifacient agent (Hosseinzadeh and Nassiri-Asl, 2013 ▶; Schmidt et al., 2007 ▶). Farmer women exposed to saffron had increased rates of miscarriage (Ajam et al., 2014 ▶). This could be due to the fact that saffron stimulates uterine contractions. However, in Table 1 of the article by Mokhtari-Zaer et al. (2015) ▶, a relaxant effect of saffron on uterine contraction was mentioned (Mokhtari-Zaer et al., 2015 ▶). Concerning various components of saffron including crocin, picrocrocin, crocetin, and safranal, each could have different actions on muscular tissue. As an antispasmodic, saffron is used for stomach pain by helping digestion and improving appetite. It also reduces tension and alleviates symptoms of premenstrual syndrome and renal colic (Agha-Hosseini et al., 2008 ▶; Moshiri et al., 2006 ▶; Kashani et al., 2018 ▶). In one study, the impact of saffron on cervical ripening in term pregnant women was evaluated for the first time in the form of a clinical trial (Sadi et al., 2016 ▶). They concluded that saffron could induce cervical priming which is a perquisite for vaginal delivery by improving the bishop score. Cervical ripening caused by prostaglandins such as misoprostol or prostaglandin E1 is associated with simultaneous increase in uterine muscle contractility which is apparently in contrast with the effects of saffron as a relaxing agent (Vahdat et al., 2015 ▶). In a study by Sadraei et al, the effects of *Crocus sativus* extracts on uterus contractions were assessed in vitro. Following removal of the uteri of rats, spontaneous rhythmic contractions were induced using potassium chloride (KCL). According to their results, *Crocus sativus* increased these contractions and showed spasmodic action on muscle fibers (Sadraei et al., 2003 ▶). Likewise, in other studies, saffron had stimulatory effects on uterus as a result of myogenic and neurogenic actions resulting in prolonged bleeding, premature birth and abortion (Tafazoli et al., 2004 ▶; Modaghegh et al., 2008 ▶). This stimulatory effect of saffron on uterine musculature, however, is sometimes desirable in obstetrics to enhance the process of labor and also in gynecology for facilitating the surgical procedures on the uterus (Sadi et al., 2016 ▶; Vahdat et al., 2015 ▶). Thus, the exact mechanism of action of saffron derivatives on uterus and cervix has not been fully elucidated yet. In all the studies mentioned, the effects of the plant extract were assessed without purifying or differentiating its different elements which could have different or even opposing actions. With the availability of saffron tablets in Iran which mainly consists of crocin, with the brand name of Krocina, we believe that it would be much easier to assess the impact of this particular component of saffron on uterine or cervical tissue to clarify the exact mechanisms underlying abortion or induction of labor. 

In the end, we congratulate Mokhtari-Zaer et al on their article and we appreciate the Avicenna Journal of Phytomedicine editorial board for their judicious concern on this topic. We hope to read well-controlled randomized clinical trials regarding the beneficiary effects of saffron in future.

## Conflict of interest

None.
